# Prognostic value of metformin for non-small cell lung cancer patients with diabetes

**DOI:** 10.1186/s12957-018-1362-1

**Published:** 2018-03-20

**Authors:** Tongbai Xu, Dongsheng Li, Yuan He, Fuliang Zhang, Man Qiao, Yanhua Chen

**Affiliations:** Department of Respiratory Medicine, Tianjin Nankai Hospital, No.122, Sanwei Road, Nankai District, Tianjin, 300100 China

**Keywords:** Non-small cell lung cancer, Metformin treatment, Diabetes mellitus, Survival analysis

## Abstract

**Background:**

The anti-cancer role of metformin has been reported in many different kinds of solid tumors, but how it affects non-small cell lung cancer (NSCLC) is currently elusive. The aim of this study was to investigate the influence of metformin treatment on diabetic NSCLC.

**Methods:**

Two hundred fifty-five patients of diabetic NSCLC receiving therapy in our hospital from 2014 to 2016 were enrolled in our study. The information on clinical diagnosis, pathology, and prognosis as well as the influence of metformin in diabetic NSCLC were collected and assessed. Univariate and multivariate analytical techniques were applied to explore how metformin affect the survival of NSCLC.

**Results:**

One hundred fifty of the 255 diabetic NSCLC patients took metformin. The median overall survival time (OST) and disease-free survival time (DFST) were significantly prolonged with metformin treatment compared to without metformin treatment (OST 25.0 vs 11.5 months, *p* = 0.005; DFST 15.6 vs 8.5 months, *p* = 0.010). Multivariate analysis indicated that metformin treatment could be used to predict the long-term outcome of diabetic NSCLC independently (HR = 0.588, 95% CI 0.466–0.895, *p* = 0.035).

**Conclusion:**

Our study revealed that the metformin could help in improving the final outcome of NSCLC patients with diabetes in the long term and thus could be applied to treat NSCLC.

## Background

The prognosis of NSCLC remains poor due to diagnosis at advanced stage and the aggressive aspect of NSCLC [1]. Although the diagnosis and therapy had been greatly improved in recent decades, the optimal prognostic factors and treatment for prolonging the survival time of NSCLC still remain to be explored, due to the complexity and heterogeneity of each NSCLC patient [2].

There were about 15–20% of all cancer patients who have diabetes mellitus at the same time [3]. Several studies demonstrated that the type 2 diabetes mellitus (T2DM) could be used independently to predict the outcome of breast cancer, pancreatic cancer, and gastric cancer [4–8]. The metformin, an oral hypoglycemic drug for T2DM, was reported to reduce the risk of cancer and cancer-related mortality in patients with diabetes mellitus [9, 10]. Recently, we explored how metformin influenced clinic pathological progress and prognosis of small cell lung cancer (SCLC), found that metformin significantly reduced the recurrence rate of SCLC (*p* = 0.001), and also demonstrated that metformin could improve the prognosis of SCLC independently [11]. Moreover, some clinical and experimental studies confirmed that metformin had the anti-tumor effect and could prolong the survival time of patients who had been diagnosed of diabetes mellitus accompanied by hepatocellular carcinoma or prostate cancer in a long term [10, 12, 13].

To date, there was few clinical trial or clinical data about the influence of metformin on the outcome of diabetic NSCLC. Thus, our study aimed at exploring how metformin affect clinical properties of a large amount of NSCLC patients, as well as the role of metformin in prognosis of diabetic NSCLC.

## Methods

This retrospective study was conducted in the hospital of Nankai, Tianjin, from February 2010 to November 2016. Two hundred fifty-five patients diagnosed with NSCLC and pre-existing T2DM were enrolled in the study, and the diagnosis of NSCLC was based on the histological examination. The study was approved by the ethnic committee of Nankai Hospital, Tianjin. Informed agreement was signed by all the enrolled patients or their relatives. Patients were excluded if (1) the information of clinical therapy or the follow-up was not complete, (2) the metastasis was found in distance or on peritoneum during the surgery, (3) they had type 1 diabetes mellitus, and (4) they received both metformin and insulin treatment.

The clinical information, pathological variables, and therapeutic strategies were all collected and assessed. According to the guideline of Response Evaluation Criteria in Solid Tumors, the response evaluation was performed every two cycles. The follow-up result was obtained by clinical visit or family contact. The OST was defined as the time from the date of diagnosis to that of death or the last follow-up visit.

### Statistical analysis

SPSS version 19.0 was used to perform the statistical analyses. The *χ*^2^ test was applied to assess the relationship between metformin treatment and clinical properties. The Kaplan–Meier curves and multivariate analyses were conducted to analyze the outcome and to identify the independent prognostic factors. G Power software [14] was used to perform power analysis for sample size, making sure that enough samples were enrolled in the current study. *p* values (two sides) < 0.05 was considered statistically significant.

## Results

### Clinic pathological features

These patients (Table 1) were divided into two groups, group A: metformin treatment and group B: non-metformin treatment. One hundred fifty (58.8%) patients were included in group A. The metformin dosage changes from 500 mg two times daily (bid) to 1000 mg two times daily for optimal effects. Power analysis using G Power software demonstrated that patient number in current study had adequate power to detect statistical significance (power = 96.6%, type I/II error rate α was 0.05). The main histology (*n* = 204; 80.0%) of lesion in included patients was non-squamous. Thirty (35.3%) patients experienced a recurrence. There was no significant difference in hemoglobin A1c (HbA1c) between these two groups (Table 1). Among all the patients, a total of 183 (72.8%) patients received chemotherapy: 105 (70.0%) in group A and 78 (75.3%) in group B. The chemotherapy plan contains carboplatin (Paraplatin) or cisplatin (Platinol) accompanied with docetaxel (Taxotere) and gemcitabine (Gemzar). All the patients also kept taking metformin during the chemotherapy. The median time of following up these patients was 67.0 months.

**Table 1:** Correlations between metformin use and clinic pathological characteristics in diabetic NSCLC patients (*n* = 255)

	All (*n* = 255)	Metformin (*n* = 150, 58.8%)	Non-metformin (*n* = 105, 41.2%)	*p* value
Age (years)				
≥ 65	117 (45.9%)	63 (42.0%)	54 (51.4%)	0.565
< 65	138 (54.1%)	87 (58.0%)	51 (48.6%)	
Gender (%)				
Male	204 (80.0%)	108 (72.0%)	96 (91.4%)	0.053
Female	51 (20.0%)	42 (28.0%)	9 (8.6%)	
Smoking history				
No	21 (8.2%)	12 (8.0%)	9 (8.6%)	0.612
Yes	234 (91.8%)	138 (92.0%)	96 (91.4%)	
BA1c (mmol/mol)		51.7 ± 14.5	53.3 ± 18.65	0.238
Histology				
Squamous	51 (20.0%)	24 (16.0%)	27 (15.7%)	0.357
Non-squamous	204 (80.0%)	126 (84.0%)	78 (74.3%)	
Tumor location				0.189
Left	114 (54.1%)	63 (42.0%)	51 (48.6%)	
Right	141 (55.3%)	87 (58.0%)	54 (51.4%)	
Stage				
I	69 (27.1%)	42 (28.0%)	27 (25.7%)	0.541
II	144 (56.5%)	87 (58.0%)	57 (54.3%)	
III	42 (16.4%)	21 (14.0%)	21 (20.0%)	
Chemotherapy				
No	72 (28.2%)	45 (30.0%)	27 (25.7%)	0.200
Yes	183 (72.8%)	105 (70.0%)	78 (75.3%)	
Thoracic irradiation				
No	213 (83.6%)	126 (84.0%)	87 (82.9%)	0.554
Yes	42 (16.4%)	24 (16.0%)	18 (17.1%)	
Tumor recurrence				
No	165 (64.7%)	111 (74.0%)	54 (51.5%)	0.015
Yes	90 (35.3%)	39 (26.0%)	51 (48.5%)	

### Correlation between metformin treatment and clinic pathological features

Table 2 indicated the association between the metformin treatment and clinic characteristics in diabetic NSCLC. Our study revealed that the metformin treatment significantly inhibit the tumor recurrence (*p* = 0.015) of these NSCLC patients. However, the effect of metformin treatment was not associated with patients’ gender, age, location of primary tumor, and the histology (*p* > 0.05).

**Table 2:** Univariate analysis of DFS and OS for diabetic NSCLC patients

	DFS			OS		
	*p* value	HR	95% CI	*p* value	HR	95% CI
Age (years)	0.511	1.563	1.032–1.877	0.104	1.106	0.948–1.753
< 65						
≥ 65						
Gender	0.210	0.874	0.703–1.489	0.109	0.748	0.720–1.310
Female						
Male						
Smoking status	0.355	1.575	0.998–1.981	0.647	1.171	0.598–1.301
Non-smoker						
Smoker						
Histology	0.879	0.877	0.643–1.637	0.511	0.974	0.544–1.710
Squamous						
Non-squamous						
Tumor location	0.105	0.987	0.774–1.021	0.314	1.014	0.784–1.879
Left						
Right						
Stage	0.001	1.878	1.170–2.474	0.037	1.440	1.110–1.901
I						
II						
III						
Tumor recurrence	0.513	1.547	1.104–1.987	0.010	1.754	1.245–2.014
No						
Yes						
Metformin use	0.010	0.69	0.387–0.947	0.005	0.548	0.401–0.755
No						
Yes						

### Survival analysis for diabetic NSCLC patients

**Fig. 1:**
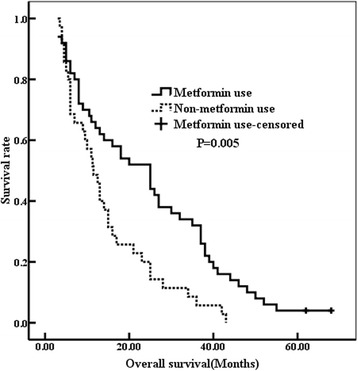
The overall survival was improved in the metformin use group (25.0 months vs 11.5 months, *p* = 0.005)

**Fig. 2:**
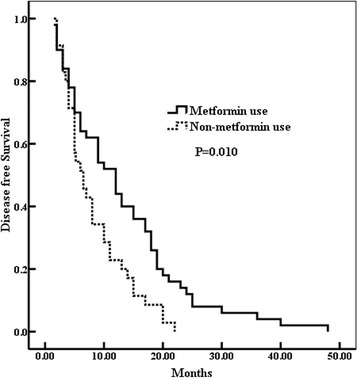
The patients in the metformin use group had prolonged disease-free survival (DFS) (*p* = 0.010)

As Fig. 1 indicated, the overall survival time (OST) of the diabetic NSCLC patients in group A was longer than that in group B (OST 25.0 and 11.5 months relatively, *p* = 0.005). In addition, disease-free survival time (DFST) of the diabetic NSCLC patients in group A is longer than that in group B (*p* = 0.010; Fig. 2). Therefore, metformin treatment can improve the prognosis of diabetic NSCLC.

Univariate analysis showed that the stage of tumor (*p* = 0.037), tumor recurrence (*p* = 0.010), and metformin treatment (*p* = 0.005) could significantly affect OST. Moreover, the tumor stage (*p* = 0.001) and metformin treatment (*p* = 0.010) could be used as the prognosis indicator of DFS (Table 2). The further multivariate analysis indicated that the tumor stage (HR = 1.758, *p* = 0.022) and metformin treatment (HR = 0.687, *p* = 0.011) could be used as independent factors for DFS (Table 3). The tumor stage (HR = 1.510, *p* = 0.008), tumor recurrence (HR = 1.700, *p* = 0.017), and metformin treatment (HR = 0.588, *p* = 0.035) were confirmed to predict the prognosis of OS independently (Table 3).

**Table 3:** Multivariate analysis for DFS and OS of diabetic NSCLC patients

	DFS			OS		
	*p* value	HR	95% CI	*p* value	HR	95% CI
Stage	0.022	1.758	1.157–2.304	0.008	1.510	1.224–1.987
I						
II						
III						
Tumor recurrence	–	–	–	0.017	1.700	1.311–2.247
No						
Yes						
Metformin use	0.011	0.687	0.414–0.903	0.035	0.588	0.466–0.895
No						
Yes						

## Discussion

Lung cancer is the most common causes of cancer death worldwide [15]. As one subgroup of lung cancers, NSCLC represents about 80% of all lung cancers diagnosed [1]. Most NSCLC patients already present with advanced stages at initial diagnosis, metastasis, and early relapse. Moreover, the prognosis of NSCLC remains unsatisfactory [15]. Therefore, it is necessary to find more sensitive NSCLC biomarkers and effective novel drugs to prolong the OST of NSCLC. In our study, metformin treatment were found to inhibit the tumor recurrence (*p* = 0.001) and present a beneficial prognosis of diabetic NSCLC.

Several studies have reported that metformin was beneficial for the prognosis of several solid tumors [9–12, 16–22]. However, there were few reports about the role of metformin in diabetic NSCLC, which encouraged our study on the role of metformin in diabetic NSCLC.

From the current study, the most important finding was that the patients in group A as compared with group B were less likely to have a tumor relapse (Table 1). However, the effect of metformin treatment on diabetic NSCLC was not associated with age, gender, stage, and pathological type. These results were consistent with our previous study and other researches [22–24]. Recently, several researches reported that T2DM was more likely to induce poorer prognosis [25, 26]. Moreover, metformin improved survival benefit in NSCLC compared with other treatment agents for diabetes. Metformin treatment survival time in diabetic NSCLC patients was almost the same as that of nondiabetic people [27]. We, also found that the long-term outcome was significantly improved in group A compared to group B (OST 25.0 vs 11.5 months, *p* = 0.005; DFS 12.0 vs 6.5 months, *p* = 0.010) (Figs. 1 and 2). Multivariate Cox analysis indicated that the metformin treatment (*p* = 0.035) could be used to predict the long-term prognosis of diabetic NSCLC independently in addition to the stage (*p* = 0.008) and tumor recurrence (*p* = 0.017) (Table 3). OST was confirmed to be negatively affected by the tumor recurrence and tumor stage, but positively influenced by metformin administration. These data indicated that metformin delayed tumor progression and prolonged the survival time, thus might be a potential therapeutic candidate for diabetic NSCLC patients. These clinical data also further demonstrated that metformin might have anti-cancer properties, which were consistent with late studies. By targeting at multiple pathways or events, such as mammalian target of rapamycin, cell cycle, inflammation, glucose metabolism, angiogenesis, and cancer stem cells, metformin plays its possible anti-cancer role; among them, the AMPK activation is believed to mediate the most effects of metformin [28, 29].

This study has some limitations, which have to be presented. Firstly, some diabetes mellitus-related factors were not collected completely in this retrospective study. Secondly, the patient size in our study was relatively small, and results were needed to be further confirmed. Lastly, the molecular mechanisms of metformin’s beneficial effect on NSCLC prognosis were not clarified. In conclusion, further studies with more enrolled patients, designed with randomized clinical trials, and including molecular mechanism studies are needed in the future.

## Conclusion

Our data indicated that metformin treatment could improve the outcome of diabetic NSCLC. Thus, it might be considered as a potentially useful prognostic indicator and anti-cancer drug of diabetic NSCLC.
